# Inhibition of NADPH Oxidase 4 (NOX4) Signaling Attenuates Tuberculous Pleural Fibrosis

**DOI:** 10.3390/jcm8010116

**Published:** 2019-01-18

**Authors:** Youngmi Kim, So Yeong Park, Harry Jung, You Sun Noh, Jae Jun Lee, Ji Young Hong

**Affiliations:** 1 Institute of New frontier Research, Hallym University College of Medicine, Chuncheon 24253, Korea; Kym8389@hanmail.net (Y.K.); qkr94006@hanmail.net (S.Y.P.); harry880219@gmail.com (H.J.); nys0617@kangwon.ac.kr (Y.S.N.); iloveu59@hallym.or.kr (J.J.L.); 2 Division of Pulmonary, Allergy and Critical Care Medicine, Department of Internal Medicine, Chuncheon Sacred Heart Hospital, Hallym University Medical Center, Chuncheon 24235, Korea; 3 Lung Research Institute of Hallym University College of Medicine, Chuncheon 24253, Korea

**Keywords:** tuberculosis, fibrosis, NADPH oxidase

## Abstract

Nicotinamide adenine dinucleotide phosphate (NADPH) oxidase [NOX] enzymes serve several hemostatic and host defense functions in various lung diseases, but the role of NOX4 signaling in tuberculous pleurisy is not well understood. The role of NOX4 signaling in tuberculous pleural fibrosis was studied using invitro pleural mesothelial cell (PMC) experiments and a murine model of *Mycobacterium bovis* bacillus Calmette–Guérin (BCG) pleural infection. The production of NOX4 reactive oxygen species (NOX4–ROS) and the epithelial mesenchymal transition (EMT) in PMCs were both induced by heat-killed mycobacterium tuberculosis (HKMT). In cultured PMCs, HKMT-induced collagen-1 synthesis and EMT were blocked by pretreatment with small interfering RNA (siRNA) NOX4. Moreover, NOX4–ROS production and subsequent fibrosis were reduced by treatment with losartan and the toll-like receptor 4 (TLR4) inhibitor TAK-242. The HKMT-induced EMT and intracellular ROS production were mediated by NOX4 via the activation of extracellular signal-regulated kinase (ERK) signaling. Finally, in a BCG-induced pleurisy model, recruitment of inflammatory pleural cells, release of inflammatory cytokines, and thickened mesothelial fibrosis were attenuated by SiNOX4 compared to SiCon. Our study identified that HKMT-induced pleural fibrosis is mediated by NOX4–ERK–ROS via TLR4 and Angiotensin II receptor type1 (AT1R). There results suggest that NOX4 may be a novel therapeutic target for intervention in tuberculous pleural fibrosis.

## 1. Introduction

Tuberculosis (TB) is the ninth leading cause of death worldwide and the leading cause from a single infectious agent [1]. Tuberculous pleurisy, the most common extrapulmonary TB, can cause exudative pleural effusions and pleural fibrosis [2]. Pleural fibrosis is particularly troubling because pleural thickening occurs in half of all patients with successfully completed treatment [3]. In severe cases, irreversible pleural fibrosis can cause chronic respiratory failure and lower a patient’s quality of life more generally [4]. Accordingly, it is worthwhile and necessary to delineate the pathogenesis of tuberculous pleural fibrosis and to ultimately conceptualize novel therapeutic targets for treatment.

Fibrosis is defined as the excessive deposition of extracellular matrix (ECM) after an inflammatory condition. Oxidative stress plays a significant role in fibrosis and nicotinamide adenine dinucleotide phosphate (NADPH) oxidases [NOXs] are the main sources of reactive oxygen species (ROS) [5]. For example, NOX4 is upregulated in idiopathic pulmonary fibrosis, bleomycin-induced lung injury, and lung cancer [6,7,8,9]. Moreover, NOX4-generated ROS may mediate airway smooth muscle hypercontractility in asthma patients and alveolar capillary barrier dysfunction in patients with hyperoxia-induced lung injury [10,11]. Likewise, pleural mesothelial cells (PMCs) play an important role in pleural fibrosis and in the immune response associated with tuberculous pleurisy [4]. In fact, several studies have investigated the related signaling in PMCs in response to *Mycobacterium bovis* bacillus Calmette-Guérin (BCG) [4,12,13]. However, the role of NOX4 in tuberculous pleurisy has never been investigated.

In this study, a PMC cell model after heat-killed *M. tuberculosis* (HKMT) treatment and a BCG-induced pleurisy mouse model were used to explore the NOX4-related signaling pathway and its underlying mechanism. It was hypothesized that NOX4 activation plays a critical role in collagen synthesis and cell proliferation in PMCs after TB infection and that the inhibition of NOX4 signaling using small interfering RNA (siRNA) after TB infection may reduce pleural fibrosis.

## 2. Materials and Methods

### 2.1. Cell Lines and Animals

Human mesothelial cell, Met5A, was purchased from the American Type Culture Collection (ATCC, Manassas, VA, USA) and cultured according to the manufacturer’s instructions. Wild-type C58BL/6 mice were purchased from DooYeol Biotech (Seoul, Korea). Mice 3–4 weeks old were used to isolate the mouse pleural mesothelial cells (PMCs). Mice 8–10 weeks old, weighing 20–24 g, were used for the BCG-induced pleurisy mouse model. All animal experiments were approved by the Institutional Animal Care and Use Committee of Hallym University.

### 2.2. Isolation of Mouse Pleural Mesothelial Cells (PMCs)

Primary mouse PMCs were isolated from the diaphragm, the external surface of the heart, and lungs of mice 3–4 weeks old as previously described [13]. Pieces of diaphragm and adjunct organs were placed in a 0.25% trypsin–ethylenediaminetetraacetic acid (EDTA) solution for 1 h. After removing the intact tissues and debris, the cell suspension was centrifuged at 1500 rotations per minute for 5 min. The pellet was resuspended and maintained in Dulbecco’s modified Eagle medium (DMEM) (Gibco, Waltham, MA, USA) supplemented with 15% fetal bovine serum (FBS; Corning costar, Corning, NY, USA) and 1% penicillin/streptomycin (Sigma, St Loise, MO, USA) in a humidified incubator with 5% CO_2_ at 37 °C. The following day, the cells were washed three times with phosphate buffered saline (PBS) to remove non-adherent cells and left until they were confluent (media change every 2 days). When a homogeneous population of cobblestone PMCs was shown at passage ~3–4, the cells were subjected to experiments described below.

### 2.3. Preparation and Treatment of Heat-Killed Mycobacterium Tuberculosis (HKMT)

Heat-killed mycobacterium tuberculosis was purchased from InvivoGen (San Diego, CA, USA). The Met5A cells and mouse PMCs were treated with 10 ng/mL HKMT either with or without inhibitors for the time periods indicated. The PD98059 (MEK inhibitor), losartan (AT1R antagonist), and TAK-242 (TLR4 inhibitor) were purchased from R&D Systems (Minneapolis, MN, USA). To suppress endogenous NOX4 expression, a specific siRNA (BioneerInc, Daejeon, Korea) against NOX4 based on the target region from the *NOX4* gene (sense: 5′-UCAGACAAAUGUAGACAC-3′ and antisense: 5′-AGUGUCUACAUUUGUCUG-3′) was used in experiments. Scrambled siRNA (sense: 5′-GGTCAAGACACTATTAACA-3′ and antisense: 5′-GGATTCCTAGTGTATTTCA-3′) was used as a control (SiCon).

### 2.4. Measurement of Intracellular ROS Levels in Mesothelial Cell Lines

To measure the ROS levels of HKMT-stimulated Met5A and PMCs, 6-carboxy-2,7-dichlorodihydrofluorescein diacetate (DCFH-DA; Sigma, St Loise, MO, USA) was used according to the manufacturer’s instruction. In brief, the cells were incubated in serum-free medium with 5 μM DCFH–DA for 30 min in the dark. After washing the cells with PBS, relative fluorescence intensity was measured using confocal microscopy (Carl Zeiss LSM710).

### 2.5. BCG-Induced Pleurisy

*Mycobacterium bovis* bacillus Calmette-Guérin (BCG) was a gift from Dr. Cho (Department of Microbiology, Yonsei University College of Medicine). Mycobacterial pleurisy was generated by injecting 10^6^ CFUs BCG Pasteur in 100 µL saline into the intrapleural cavity, as previously reported [14]. Mice were monitored twice a week and sacrificed 15 days after BCG injection. The control group was injected with 100 µL sterile phosphate-buffered saline (PBS) instead of BCG into the intrapleural cavity.

Twenty-eight mice were randomly divided into four groups: PBS + SiCon (siRNA control post-treatment after PBS treatment), BCG + SiCon (SiCon post-treatment after BCG treatment), PBS + SiNOX4 (siRNA NOX4 post-treatment after PBS treatment), and BCG + SiNOX4 (siRNA NOX4 post-treatment after BCG treatment). On days of PBS or BCG injection, mice were injected intravenously with SiNOX4 or SiCon twice per week. On day 15, all mice were sacrificed and lung tissue and pleural effusion were collected.

### 2.6. Pleural Cells

Thoracic cavities from naïve and infected mice were washed with 1 mL 2 mmol/L EDTA–PBS. The pleural effusion was centrifuged (4 °C, 1500 g, 10 min) and the supernatant was used to evaluate levels of cytokines, chemokines, and CFUs. The cell pellet was reconstituted in 100 µL PBS and used to analyze the cells. Total cell numbers were counted using a homocytometer and evaluated on a cytospin with Diff-Quick staining.

### 2.7. Lung Tissue Harvest and Histopathological Analysis

The right lung was isolated and stored at −80 °C for molecular analyses after flushing the pulmonary vasculature. The left lung was excised and fixed overnight in 4% formaldehyde at 4 °C overnight. Paraffin-embedded tissues were sectioned at 5-μm thickness followed by H&E staining and Elastica van Gieson collagen staining (Millipore Corporation, Darmstadt, Germany). The changes in histology were imaged under a light microscopy. Histological lung fibrosis scores and free alveolar spaces were analyzed in accordance with previous studies [15,16].

### 2.8. Immunohistochemistry and Immunofluorescence Staining

Immunohistochemistry was performed to detect the expression level of NOX4 in lung tissues collected from mice with BCG-induced pleurisy. In brief, paraffin-embedded lung tissue sections were prepared as described above and rehydrated with a graded ethanol of 100, 70, 50, and 25%. After blocking with 5% normal goat serum (Vector laboratories, CA, USA) at room temperature for 1 h, slides were incubated with primary anti-NOX4 (1:100 dilution, Santa Cruz Biotechnology, Santa Cruz, CA, USA) at 4 °C overnight, followed by incubation with goat anti-rabbit secondary antibody for 1 h at room temperature. Immunostaining was performed with diaminobenzidine (DAB; Sigma, St Loise, MO, USA). Immunofluorescence staining was conducted to analyze co-expression of NOX4 and mesothelin in lung tissues. In brief, the sections were incubated with antibody to NOX4 (1:100 dilution, Santa Cruz Biotechnology, Santa Cruz, CA, USA) and antibody to mesothelin (1:100 dilutiob, Santa Cruz Biotechnology, Santa Cruz, CA, USA) overnight at 4 °C. After washing three times with PBS, the slides were incubated with Alexa Fluor 546-conjugated goat anti-mouse IgG1 and Alexa Fluor 488-conjugated goat anti-rabbit IgG secondary antibody for 1 h in the dark. Finally, the slides rinsed with PBS and analyzed under a fluorescent microscope (Olympus FV500; Olympus, Tokyo, Japan). DAPI (Sigma, St Loise, MO, USA) was used as counterstain.

### 2.9. Enzyme-linked Immunosorbent Assay

The presence of interferon-γ, IL-2, TNF-α, monocyte chemotactic protein (MCP) -1, and IL-6 in pleural tissue was quantified using an enzyme-linked immunosorbent assay (Cusabio, Wuhan, China).

### 2.10. Western Blotting

Western blotting was performed as described in a previous study [17]. In brief, 50 μg of proteins were separated by 10% SDS-polyacrylamide gel electrophoresis (SDS-PAGE) and transferred to PVDF membranes (Thermo Scientific, Waltham, MA, USA).Target proteins were detected with primary antibodies as follows: anti-NOX4 (Santa Cruz Biotechnology, Santa Cruz, CA, USA), anti-TLR4 (Santa Cruz Biotechnology, Santa Cruz, USA), anti-SNAIL (Santa Cruz Biotechnology, Santa Cruz, CA, USA), anti Zo-1 (Santa Cruz Biotechnology, Santa Cruz, CA, USA), anti-Collagen 1A (Santa Cruz Biotechnology, Santa Cruz, CA, USA), anti-Actin (Sigma, St Loise, MO, USA), anti-E-cadherin (Cell Signaling, Danvers, MA, USA), anti-LC3II (Cell Signaling, Danvers, MA, USA), anti-P62 (Cell Signaling, Danvers, MA, USA) and anti-phosphorylated ERK (Cell Signaling Technology, Danvers, MA, USA).HRP-conjugated goat anti-mouse and anti-rabbit IgG (Thermo Scientific, Waltham, MA, USA) were used as secondary antibodies.

### 2.11. Quantitaitve Real-Time PCR (qRT-PCR)

Total RNAs were extracted using Trizol (Invitrogen, Carlsbad, CA, USA) according to the manufacturer’s instruction. Quality and quantity of isolated RNA were detected with a NanoDrop 2000/2000c instrument (Thermo Scientific, Waltham, MA, CA, USA). The cDNA was synthesized with 5 μg of RNA using Maxime RT PreMix Kit (iNtRON Biotechnology, Seoul, Korea). The PCR reactions were performed by using the 2× Rotor-Gene SYBR 120 Green PCR Master Mix (Qiagen, Germantown, MD, USA) in the Rotor-Gene Q (Qiagen, Germantown, MD, USA). The primers were as follows: mouse collagen 1A (COL1A) (forward): 5′-CAGGGTATTGCTGGACAACGT-3′, and (reverse) 5′-GGACCTTGTTTGCCAGGTTCA-3′; human COL1A (forward): 5′-AATCCATCGGTCATGCTCTC-3′, and (reverse) 5′-ACTCGTGAGACCTGCGTGTA-3′; human Angiotensin Converting Enzyme (ACE) (forward): 5′-GCAAGGAGGCAGGCTATGAG-3′, and (reverse) 5′-CGGGTAAAACTGGAGGATGG-3′; mouse Actin (forward) 5′-AACTCCATCATGAAGTGTGA-3′ (reverse) 5′-ACTCCTGCTTGCTGATCCAC-3′; human Actin (forward) 5′-CATGTACGTTGCTATCCAGGC-3′ (reverse) 5′-CTCCTTAATGTCACGCACGA-3′.

### 2.12. Human Pleural Fluid Collection

The retrospective study was conducted at Chuncheon Sacred Heart Hospital in Korea from January 2014 to June 2016. Approval for the study was granted by the hospital’s Research Ethics Committee (institutional review board number 2012-27). Nine patients with tuberculous pleurisy and eight subjects with transudate were included in the analyses. Tuberculous pleurisy was defined by the existence of pleural fluid that was culture-positive for *M. tuberculosis* or histologically confirmed TB infection by pleural biopsy. Transudate pleural effusion was defined according to Light’s criteria [18]. The expression of NOX4 protein was measured using Western blotting and immunoprecipitation according to standard procedures [19].

### 2.13. Statistical Analyses

Statistical analyses were performed using Prism 5.1 (Graphpad software, La Jolla, CA, USA). Group comparisons were performed using unpaired *t*-test or two-way ANOVA. Data are expressed as means ± SD. of three independent experiments. *p* < 0.05 was considered to be statistically significant.

## 3. Results

### 3.1. HKMT Upregulates NOX4 Production in Human and Mouse PMCs

**Figure 1:**
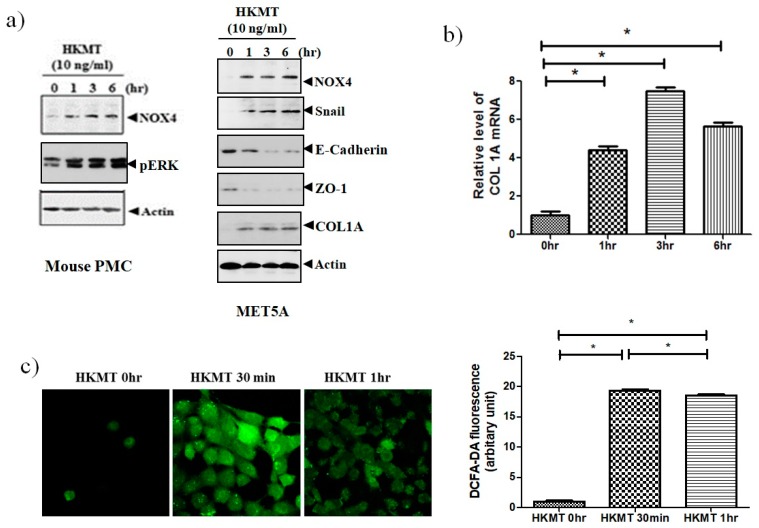
Heat-killed mycobacterium tuberculosis (HKMT) induces collagen I synthesis and NOX4–ROS expression in pleural mesothelial cells (PMC) (**a**) Western blotting analyses of NOX4, phosphorylated ERK, collagen, and EMT markers, (**b**) COL1A mRNA concentration by RT-PCR, (**c**) representative photomicrographs of cells subjected to 6-carboxy-2’,7’-dichloroflurescin diacetate (DCFA-DA) treatment. * *p* < 0.01 vs. 0 h after HKMT treatment. Data are presented as the mean ± SD of three independent experiments. NOX, NADPH oxidase; EMT, epithelial to mesenchymal transition; COL1A, Collagen 1A; ROS, reactive oxygen species.

To study of the effect of HKMT on NOX4 production, mouse PMCs and human Met5A cells were incubated with HKMT (10 ng/mL) for 1–6 h. As shown in Figure 1, HKMT treatment increased the expression of NOX4 protein and the production of collagen RNA and protein. Mesothelial cells undergo epithelial to mesenchymal transition (EMT) in the process of tuberculous pleurisy and the transcription factor snail is an inducer of EMT process [20]. The HKMT treatment upregulated snail expression and decreased the expression of epithelial markers, E-cadherin and Zo-1 protein in Met5A cells. The HKMT increased the phosphorylation of ERK 1/2 protein in PMCs. The intensity of DCFA–DA-labeling ROS in the PMCs was also markedly elevated in the HKMT group.

### 3.2. SiRNA NOX4 Interference Prevents HKMT-Induced Collagen and EMT Synthesis through the NOX4/ERK/ROS Signaling Pathway

**Figure 2:**
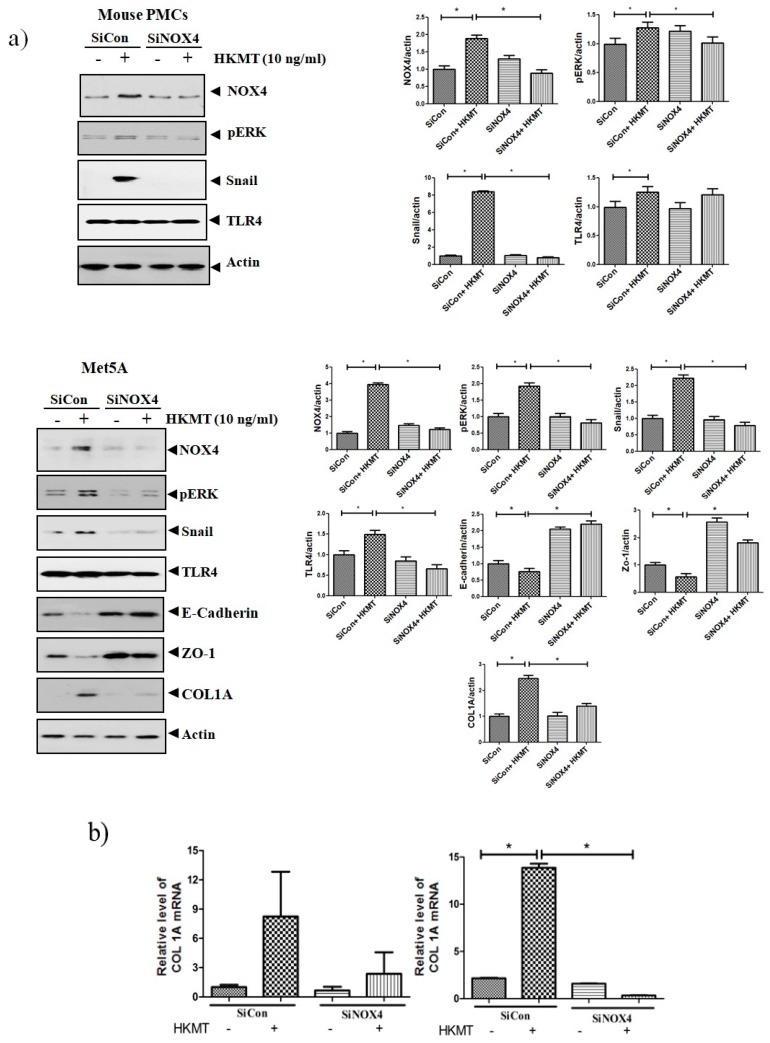

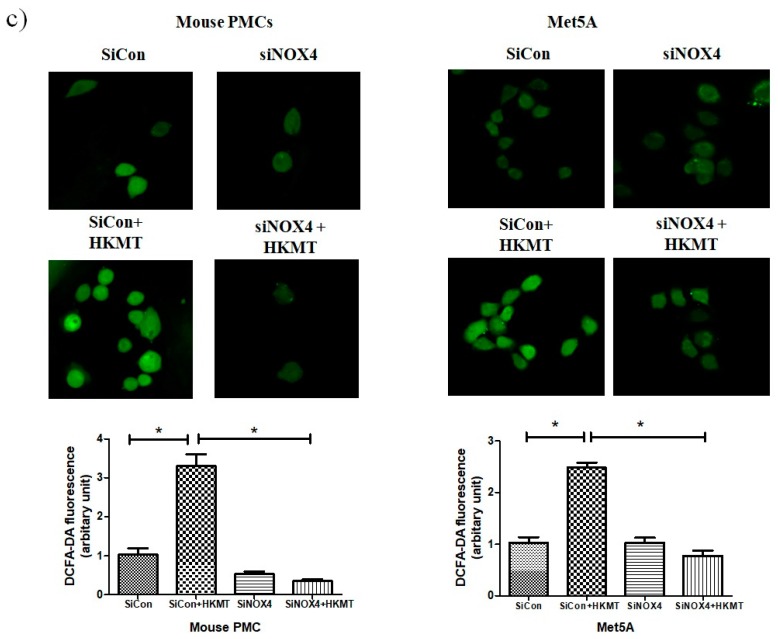
NOX4 mediates HKMT-induced collagen-1A synthesis and EMT. Pleural mesothelial cells were transfected with SiRNA targeting NOX4 (SiNOX4). Twenty-four hours later, the cells were treated with HKMT (10 ng/mL) for 1 h or were left untreated. (**a**) Western blotting analyses of NOX4, phosphorylated ERK, and collagen, and EMT markers. (**b**) COL1A mRNA level by RT-PCR. (**c**) Representative photomicrographs of cells subjected to DCFA–DA treatment. * *p* < 0.05. Data are expressed as mean ± SD. NOX, NADPH oxidase; EMT, epithelial to mesenchymal transition; COL1A, Collagen 1A. Data are presented as the mean ± SD of three independent experiments.

To further study the role of NOX4 in HKMT-induced collagen increase, siRNA NOX4 was used to block NOX4 in PMCs. As depicted in Figure 2, siRNA NOX4 treatment blocked HKMT-induced collagen synthesis and expression of phosphorylated ERK. Also, SiRNA NOX4 treatment reversed the EMT process as downregulation of Snail expression and upregulation of E-cadherin and Zo-1 protein. Moreover, the intensity of DCFA–DA labeled ROS in the cells was significantly reduced in the siRNA NOX4 + HKMT group than in the HKMT group (Figure 2c). These findings suggest that HKMT induces collagen synthesis by activating NOX4–ERK–ROS signaling.

**Figure 3:**
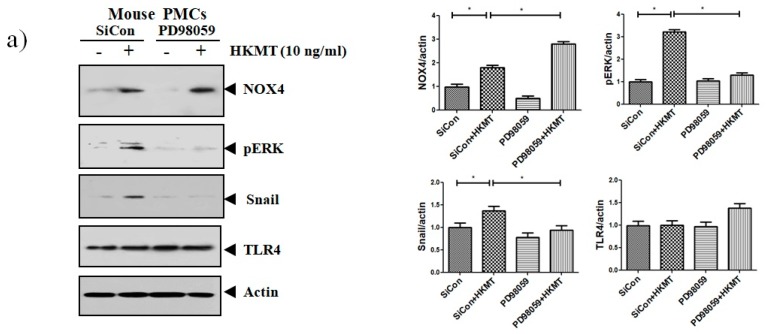

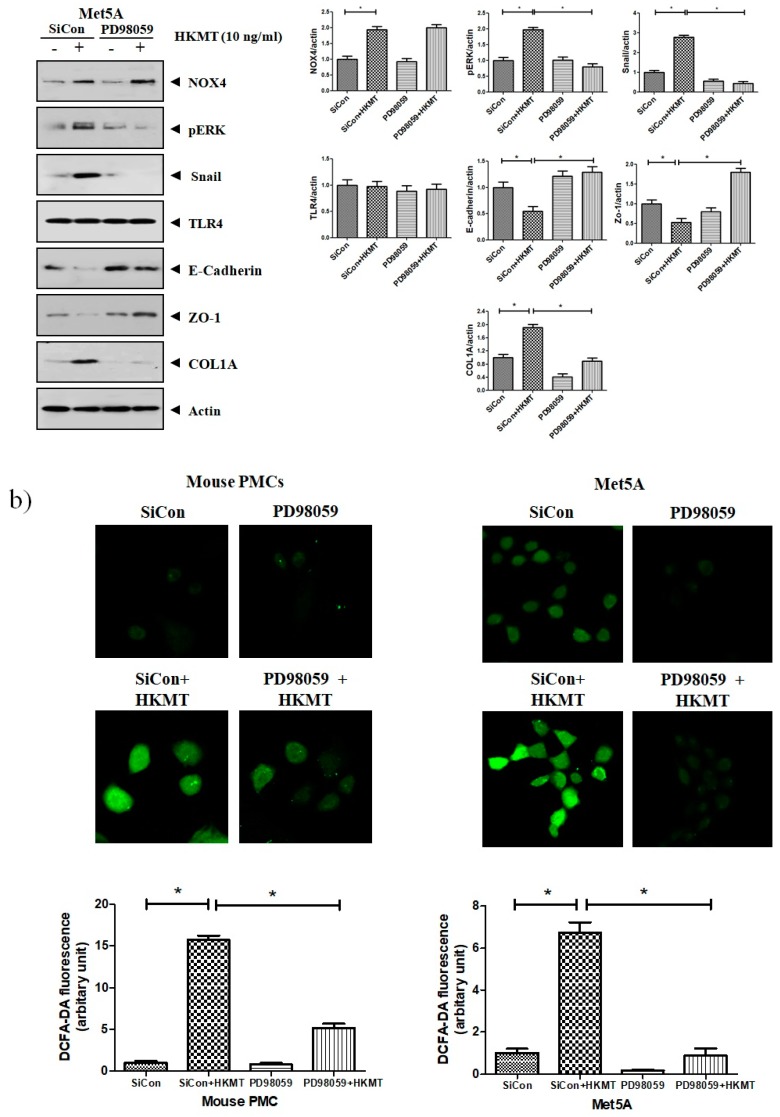
NOX4 mediates HKMT-induced EMT and intracellular ROS production via activation ERK signaling. PMCs were treated with HKMT in the presence or absence of the ERK inhibitor (PD98059). (**a**) Western blotting analyses of NOX4, phosphorylated ERK, collagen, and EMT markers. (**b**) Representative photomicrographs of cells subjected to DCFA–DA treatment * *p* < 0.05. Data are presented as the mean ± SD of three independent experiments. NOX, NADPH oxidase; EMT, epithelial to mesenchymal transition; COL1A, Collagen 1A.

Accordingly, to clarify the signaling-mediating NOX4 expression in HKMT-stimulated PMCs, the signaling pathway was explored via pretreatment with MEK inhibitor (PD98059, 10 μM). As depicted in Figure 3, the expression of collagen synthesis and Snail were markedly attenuated by pretreatment with MEK inhibitor, but the expression of NOX4 protein was not. These findings demonstrate that HKMT induces NOX4-dependent ERK–ROS pathway in human and mouse PMCs.

### 3.3. ANG II Type I Receptor and TLR4 are Involved in HKMT-Induced NOX4-ROS Signaling and Collagen Synthesis

**Figure 4:**
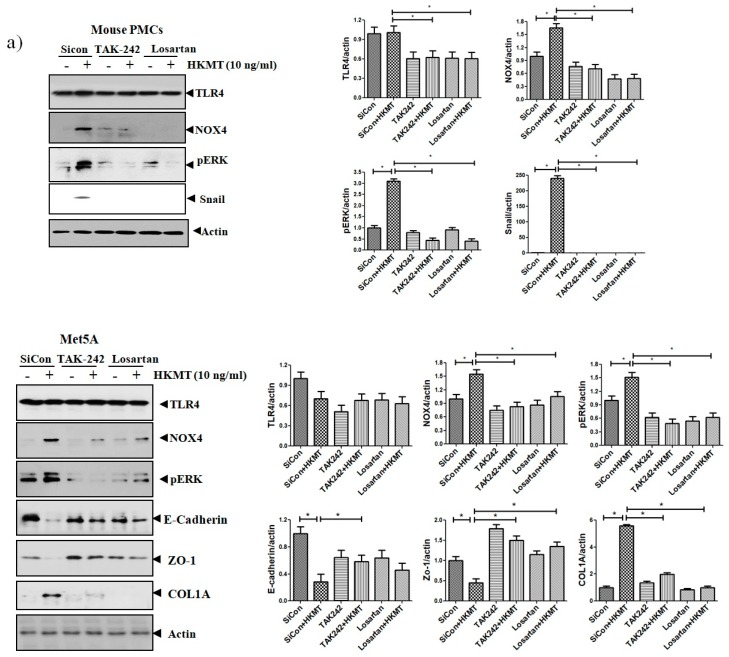

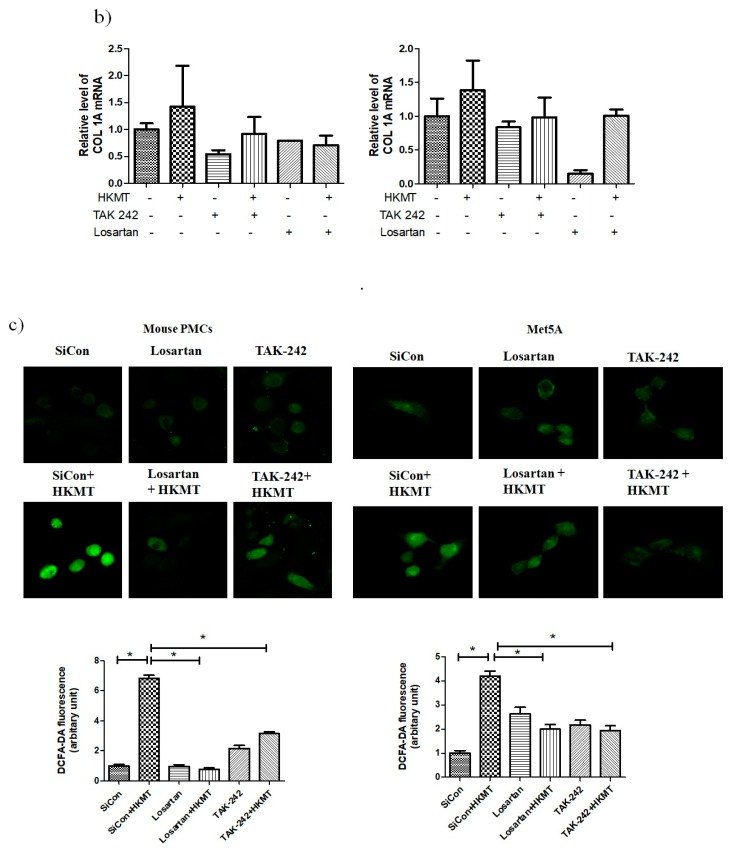
Toll like receptor 4 (TLR4) inhibition and angiotensin II receptor type1 (AT1R) inhibition reduce HKMT-induced NOX–ROS production and collagen synthesis. (**a**) Western blotting analyses of NOX4, phosphorylated ERK, collagen, and EMT markers. (**b**) Collagen-1 mRNA concentration by RT-PCR. (**c**) Representative photomicrographs of cells subjected to DCFA–DA treatment. Data are presented as the mean ± SD of three independent experiments.* *p* < 0.05. Losartan, AT1 receptor antagonist; TAK242, NOX: NADPH oxidase; EMT, epithelial to mesenchymal transition; COL1A, Collagen 1A.

The binding of Ang II to its receptor is associated with intracellular free radical generation and tissue damage [21]. Several studies have suggested that NOX4 plays a critical role in the TLR4 downstream signaling cascade [22,23]. We studied the role of Ang II type 1 receptor (AT1R) and TLR4 in NOX4-mediated collagen synthesis in PMCs after HKMT treatment. Losartan (an AT1R antagonist) and TAK-242 (a TLR4 inhibitor) prevented the HKMT-induced increase in NOX4 and collagen synthesis in mouse PMCs and human Met5A cells (Figure 4a,b). Similarly, both decreased the levels of HKMT-induced DCFA DA-labeled ROS and reversed the EMT process (Figure 4c).

These findings suggest that NOX4-induced collagen synthesis and ROS production after HKMT treatment were carried out by AT1R and TLR4. The decrease in LC3II (autophagosome formation) and the buildup of P62 (sequestosome-I) after HKMT treatment were also reversed by both losartan and TAK-242 (included in Figure S1).

### 3.4. Crosstalk between the AT1 Receptor and TLR4 in PMCs Contributes to HKMT-Induced ROS Production

**Figure 5:**
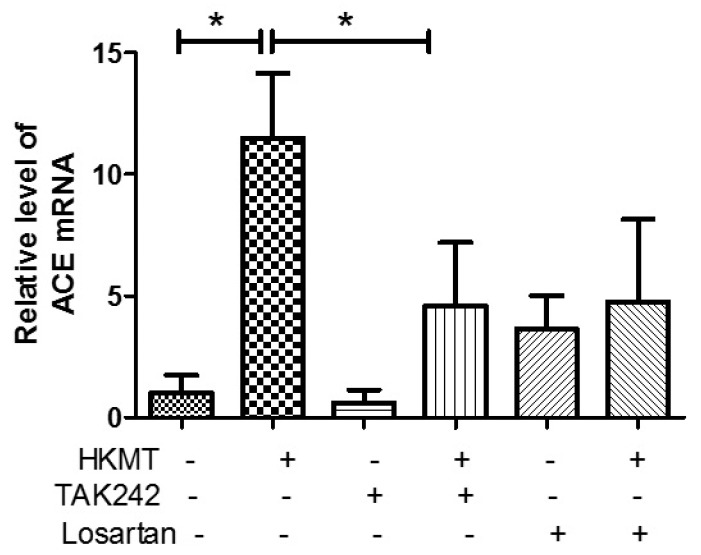
Upregulates angiotensin converting enzyme (ACE) expression after HKMT treatment. ACE mRNA concentration by RT-PCR. Data are presented as the mean ± SD of three independent experiments.* *p* < 0.05. Losartan: AT1 receptor antagonist; TAK242: TLR4 receptor antagonist.

Angiotensin converting enzyme (ACE) cleaves ANG I to form ANG II, which serves as the ligand for AT1R. In this study, we demonstrated that TAK-242 decreased HKMT-induced ACE mRNA expression (Figure 5). Toll like receptor 4 (TLR4) is involved in upregulating ACE levels after HKMT treatment leading to enhance ANG II-mediated ROS production. Taken together, these findings suggest that there are functional interactions between AT1R and TLR4 in PMCs exposed to HKMT.

### 3.5. BCG-Induced Pleurisy Increases the Expression of Lung NOX4 and NOX4 Antagonism Attenuates Changes in the Tissue in BCG-Induced Pleurisy

**Figure 6:**
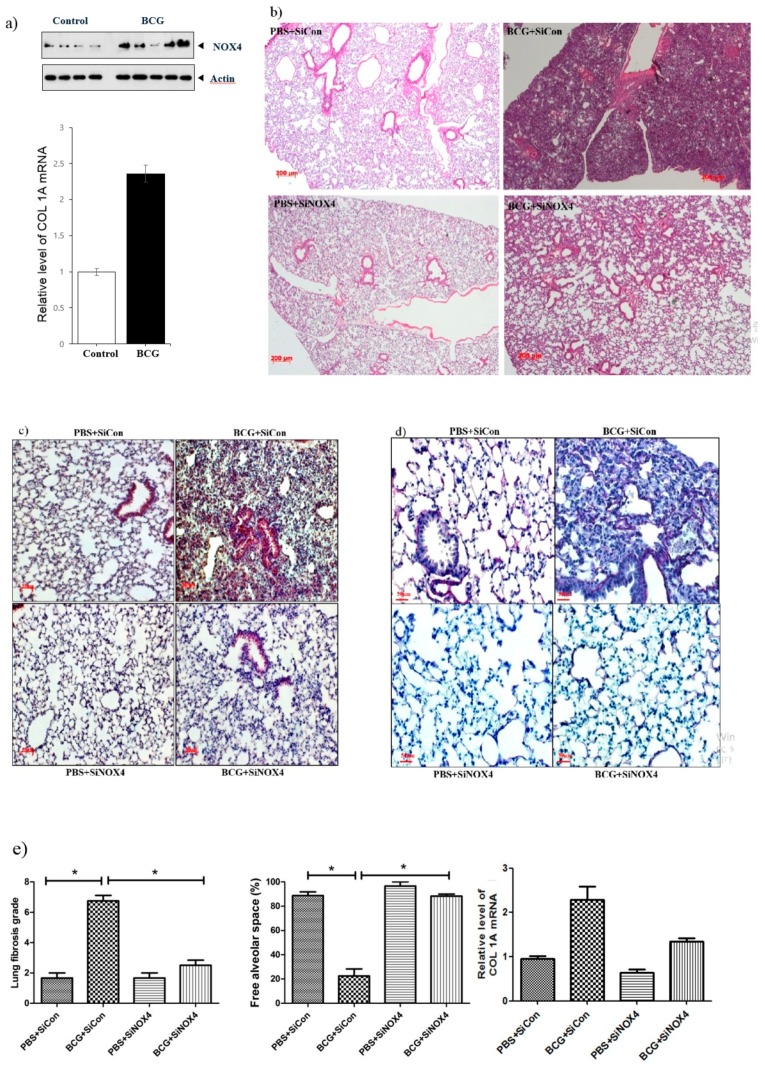
NOX4 levels increase in BCG-induced pleurisy lung fibrosis. SiNOX4 post-treatment attenuates the hyperplasia of the visceral pleura, granuloma, and bronchiolitis induced by BCG in the mouse model. (**a**) Western blotting analyses of NOX4 and Collagen-1A mRNA concentration by RT-PCR. (**b**) Microscopic examination of lungs at days15 in four groups (PBS + SiCon group, BCG + SiCongroup, PBS + SiNOX4 group, and BCG + SiNOX4 group). Seven mice per group were analyzed. Scale bar: 200 μm. (**c**) Immunostaining of NOX4 in mouse lungs. Scale bar: 50 μm. (**d**) Immunostaining of Elastica van Gieson in mouse lungs. (Collagen: red color). Scale bar: 50 μm. (**e**) Quantification of lung fibrosis and free alveolar space. * *p* < 0.05. BCG, *Mycobacterium bovis* bacillus Calmette-Guérin; PBS, phosphate buffered saline; COL1A, collagen 1A.

In an in vivo experiment, a BCG-induced pleurisy mouse model was made consisting of four groups: PBS + SiCon (*n* = 7), BCG + SiCon (*n* = 7), PBS + SiNOX4 (*n* = 7), and BCG + SiNOX4 (*n* = 7). The expression of NOX4 protein and collagen synthesis in lung tissue increased following BCG injection compared to PBS treatment (Figure 6a). Fifteen days after BCG infection, all mice in the BCG + SiCon group presented with granulomas with increased pleural hyperplasia and enlarged submesothelia. Mice in the BCG + SiNOX4 group showed decreased pleural hyperplasia and pulmonary cell infiltration compared to mice in the BCG + SiCon group (Figure 6b). Immunostaining of the lung tissue of mice for NOX4 showed faintly detectable expression in epithelial bronchial cells in the PBS + SiCon group. While mice in the BCG + SiCon group showed stronger increases in NOX4 around inflamed areas with thickened septae, mice in the BCG + SiNOX4 group showed reduced NOX4 expression in lung tissue (Figure 6c). There were also deposits of collagen in the pleural sections of mice in the BCG + SiCon group but mice in the BCG + SiNOX4 group had fewer collagen deposits compared to mice in the BCG + SiCon group (Figure 6d).

A histologic examination of the lungs of mice that received BCG + SiNOX4 treatment revealed a lower grade of lung fibrosis (BCG + SiNOX4: 2.5 ± 0.8, BCG + SiCon: 6.7 ± 1.0, *p* < 0.001) and a larger free alveolar space (BCG + SiNOX4: 88.3 ± 4.1, BCG + SiCon: 22.5 ± 16.0, *p* < 0.001) compared to mice that received the BCG + SiCon treatment (Figure 6e). In addition to the histological findings, RT-PCR indicated that collagen mRNA levels were reduced in mice in the BCG + SiNOX4 group compared to those in the BCG + SiCon group.

### 3.6. Activation of NOX4 in the Mesothelium in BCG-Induced Pleurisy

**Figure 7:**
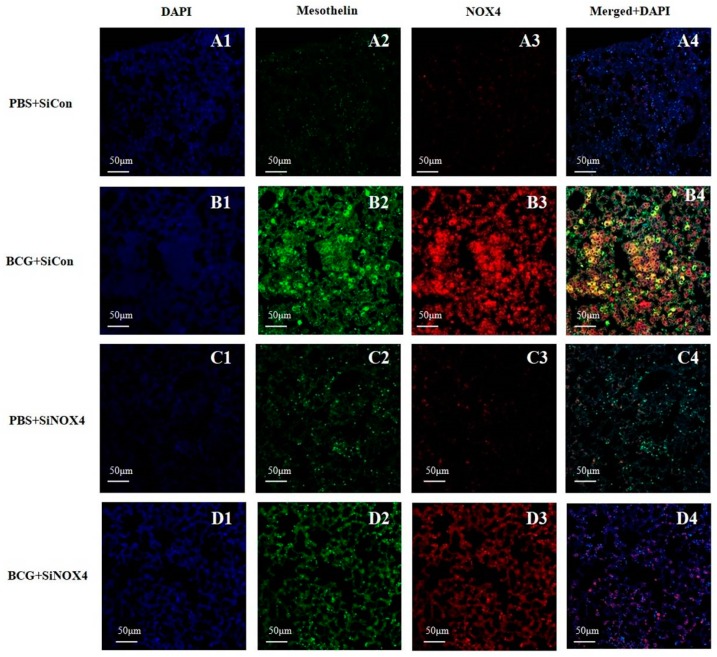
Activation of NOX4 and mesothelin deposition in a BCG-induced pleurisy mouse model. NOX4 and mesothelin (markers for PMCs) were subjected to immunofluorescence staining. **A1**–**D1**: DAPI staining. **A2**–**D2**: Immunofluorescence staining of mesothelin (green color). **A3**–**D3**: Immunofluorescence staining of NOX4 (red color). **A4**–**D4**: Overlays of immunofluorescence staining and DAPI staining, yellow areas represent colocalization of mesothelin and NOX4. DAPI, 4′,6-Diamidino-2-phenylindole dihydrochloride.

Lung immunostaining of mesothelin can be used to detect mesothelial cells. As depicted in Figure 7, pleural sections contained a very high level of NOX4 that was colocalized with mesothelin, a marker of mesothelial cells in the BCG + SiCon group. The inhibition of NOX4 signaling by SiNOX4 post-treatment blocked the BCG-induced mesothelin expression in lung tissue. This indicates that NOX4 increased in the PMCs of mice with BCG-induced pleurisy as per the cell experiment.

### 3.7. NOX4 Antagonism Reduces Cell Accumulation and the Release of Inflammatory Cytokines in the Pleural Cavity in BCG-Induced Pleurisy

**Figure 8:**
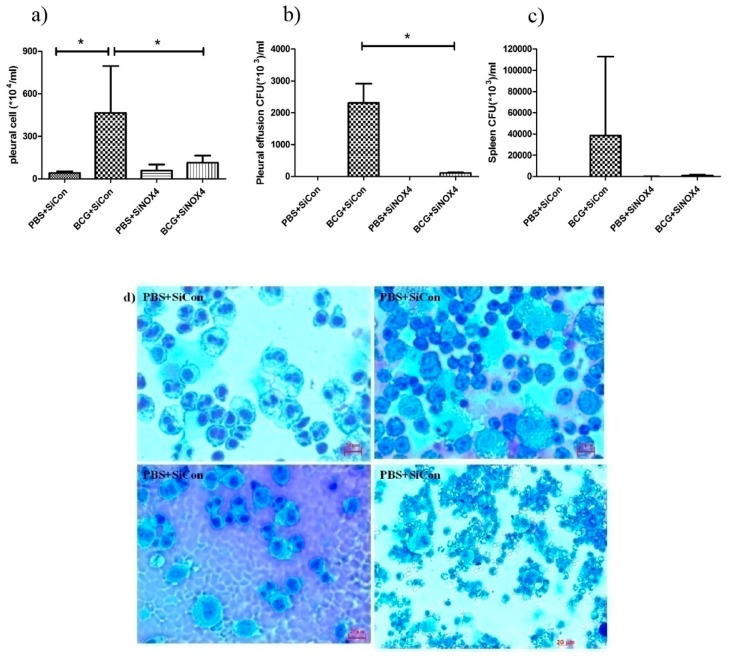

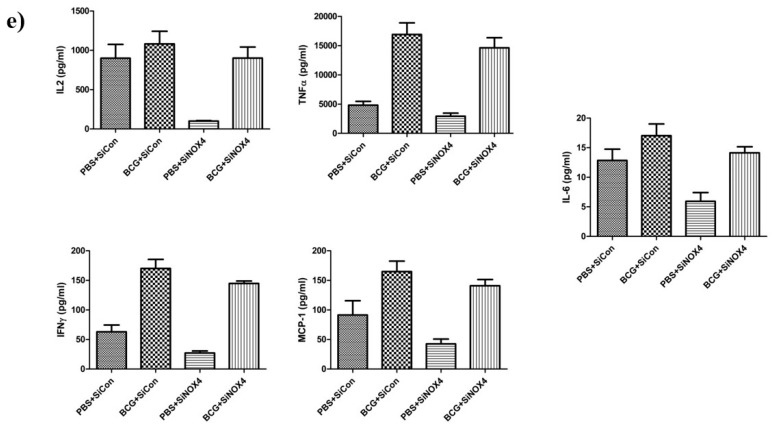
BCG-induced pleurisy and accumulation of cells in the pleural cavity. (**a**) Total number of cells in the pleural cavities in four groups (PBS + SiCon group, BCG + SiCon group, PBS + SiNOX4 group, and BCG + SiNOX4 group) Seven mice per group were analyzed. (**b**) Bacterial load in pleural effusion in four groups. (**c**) Bacterial load in spleen in four groups. (**d**) Photomicrographs from cytospin stained with Giemsa. (**e**) Cytokine levels (Interferon-ɤ, IL-2 TNF-α, MCP-1, and IL-6) were assessed in the pleural fluid. * *p* < 0.05.

To examine the effects of siRNA NOX4 post-treatment in BCG-induced pleurisy, cell recruitment and the accumulation of cytokines were determined. On day 15 after infection, there was a significant increase in the total number of pleural cells (PBS + SiCon: 40.5 × 10^4^ ± 11.6 × 10^4^/mL, BCG + SiCon: 465.0 × 10^4^ ± 331.1 × 10^4^/mL, *p* = 0.034) (Figure 8a). The total count in mice in the BCG + SiNOX4 group was significantly lower than that in the BCG + SiCon group (BCG + SiNOX4: 113.4 × 10^4^ ± 51.2 × 10^4^/mL, *p* = 0.043). We determined the bacterial load in the pleural fluid and spleen. The BCG + SiCon group exhibited substantial bacterial loads in the pleural fluid on day 15 (2313.0 × 10^3^ ± 600.0 × 10^3^ CFU/mL) and BCG + SiNOX4 group depicted significantly lower bacterial loads in the pleural fluid than BCG + SiCon group (BCG + SiNOX4: 110.0 × 10^3^ ± 14.1 × 10^3^ CFU/mL, *p* = 0.016, Figure 8b).

The amount of CFUs in spleens was relatively lower in the BCG + SiNOX4 group compared to the BCG + SiCon group (BCG + SiNOX4: 38460 × 10^3^ ± 74420 × 10^3^ CFU/mL, BCG + SiCon: 947.6 × 10^3^ ± 702.4 × 10^3^ CFU/mL, *p* = 0.427) (Figure 8c). Microscopic analyses of pleural cells revealed multinucleated giant macrophages in mice in both the BCG + SiCon group and the BCG + SiNOX4 group (Figure 8d). The concentration of cytokines (IL-6, TNFα, IFNɤ, MCP-1, IL-2) in pleural effusion were higher in mice in the BCG + SiCon group than in the PBS + SiCon group, which is consistent with previous studies. A lower expression of cytokines was detected in the pleural effusion of mice in the BCG + SiNOX4 group compared to the BCG + SiCon group (Figure 8e). These results suggest that NOX4 antagonism leads to substantial control of pleural inflammation and infection.

### 3.8. BCG Upregulates the Expression of ERK and Downregulates Autophagy Signaling via NOX4 Signaling

**Figure 9:**
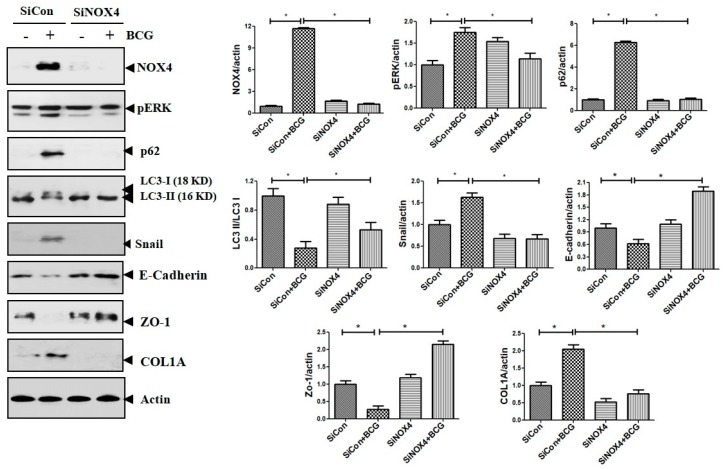
The effect of SiRNA post treatment in BCG-induced pleurisy model. The expression of NOX4, phosphorylated ERK, LC3II, P62, collagen, and EMT markers in mouse lung lysates after BCG infection and SiNOX4 post-treatment.

The findings also suggest that NOX4–ERK–ROS signaling is involved in TB-induced EMT processes in human and mouse cell experiments. Consistent results were found in mouse models. As depicted in Figure 9, compared to the PBS + SiCon group, mice in the BCG + SiCon group showed significantly higher expressions of ERK phosphorylation and Snail. In mice in the BCG + SiNOX4 group, the expression of ERK phosphorylation and Snail remarkably decreased than BCG + SiCon group. The expression of LC3II decreased and the expression of P62 in lung tissue was higher in mice in the BCG + SiCon group than in the PBS + SiCon group. The SiNOX4 post-treatment blocked the downregulation of LC3II and upregulation of P62. These results suggest that BCG-induced NOX4 expression may regulate autophagy signaling.

### 3.9. Expression of NOX4 in Human Pleural Effusion is Upregulated in Patients with Tuberculous Pleurisy

The NOX4 protein expression was measured in pleural effusion in human adults by Western blotting and immunoprecipitation (Figure S2). While the expression of NOX4 protein was remarkable in pleural effusions of adults with tuberculous pleurisy, it was undetected in pleural effusions with adults with transudate.

## 4. Discussion

Nicotinamide adenine dinucleotide phosphate (NADPH) oxidase [NOX] enzymes play an important role in generating ROS [24]. They participate in signal transduction, angiogenesis, and a variety of immune responses [25,26], and contribute to the pathogenesis of several lung diseases [6,7,8,9,10,11,24,27]. For example, NOX2 modulates inflammatory responses in asthma, while NOX4 mediates airway smooth muscle hypercontractility [10]. In idiopathic pulmonary fibrosis, NOX4 also mediates TGF-β-induced fibroblast differentiation into myofibroblasts [8] and contributes to myofibroblast differentiation by activating multiple signal pathways such as Smad 2/3 phosphorylation, JNK, and P38MAP kinase [7]. NADPH oxidase 2 (NOX2) and NOX4 play distinct roles in regulating lung inflammation and apoptosis, as well as permeability in *Pseudomonas aeruginosa* lung infection [27].

In the present study, the mechanisms underlying the fibrosis associated with tuberculous pleurisy were explored. For the first time, it was found that NOX4 plays an important role in tuberculous pleural fibrosis. The in vitro data showed that HKMT induces NOX4 activation and EMT in human Met5A cells and mouse PMCs, and that inhibition of NOX4 prevents HKMT-induced collagen synthesis and fibrosis. HKMT upregulated phosphorylated ERK expression in human Met5A cells and mouse PMCs through the activation of NOX4 signaling. The activation of NOX4 and the subsequent triggering of the ERK/ROS/EMT signaling pathway resulted from both the ACE-Ang II system and TLR 4 activation. Angiotensin converting enzyme (ACE) and Ang II are associated with tissue remodeling in fibrotic diseases such as cardiovascular fibrosis and pulmonary fibrosis [28,29]. Ang II type I receptor participates in TPE-induced collagen synthesis in PMCs [4]. Mesothelial cells produce various toll-like receptors in response to endotoxins and MTB infection [13,30,31].

We also found that blocking ATR and TLR4 using losartan and TAK-242 inhibits HKMT-induced NOX4 expression and subsequent ERK–ROS signaling. These results are consistent with previous studies. For example, Zhang et al showed that NOXs has two time-dependent reactions in response to Ang stimulation via MAPK pathways [32]. Angiotensin plays a critical role in lung fibrosis via activation of NOX-mediated damage [33,34]. NADPH oxidase 4 (NOX4) plays a role in TLR4 signaling cascades under different stimuli such as LPS, insulin, and angiotensin II [35]. Nakashima et al. reported that TLR4 plays a critical role in regulating Ang II induced-ROS levels by activating NOX4 in vascular remodeling [23]. Interestingly, the present research supports crosstalk between TLR4 and AT1R in tuberculous pleurisy cell models. Blocking the TLR4 receptor before HKMT treatment decreased the level of ACE, the enzyme that produces Ang II (the ligand of AT1R).

*Mycobacterium bovis* bacillus Calmette-Guérin (BCG) induced pleurisy in a murine model confirmed the role of NOX4 signaling in tuberculous fibrosis in accordance with in vitro PMC experiments. Post-treatment with SiNOX4 attenuated hyperplasia of visceral pleura, decreased free alveolar spaces, and increased pulmonary cell infiltration caused by BCG injection. We also explored BCG-induced pleurisy cell recruitment and infected phagocytes as reported in other animal studies. Cytospin preparation with pleural cells showed that SiNOX4 treatment after BCG injection did not prevent the production of phagocytes or the capacity to eliminate bacilli. Antagonizing the NOX4 receptor signaling during BCG-induced pleurisy decreased pleural inflammation and BCG bacterial load.

Antagonizing NOX4 expression after BCG exposure resulted in downregulation of the expression of ERK–ROS signaling akin to in vitro PMC experiments. Moreover, the results suggested that NOX4 signaling may be involved in impaired autophagy in the BCG-induced pleurisy model. Autophagy controls organelle quality by degrading dysfunctional cellular organelles [36]. Several previous studies have evaluated the clinical relevance of autophagy in TB infection and the protective role of autophagy in bleomycin and cigarette-related lung fibrosis [34,37,38,39]. The protective effects of Ang (1–7) against bleomycin and cigarette-related lung fibrosis through inhibition NOX4-derived ROS is consistent with our finding that losartan and TAK-242 improved autophagy caused by NOX-ROS signaling in TB [34,39]. However, exploring the possible mechanism of NOX4 in autophagy inhibition in tuberculous pleurisy is still needed to identify the potential therapeutic target.

**Figure 10:**
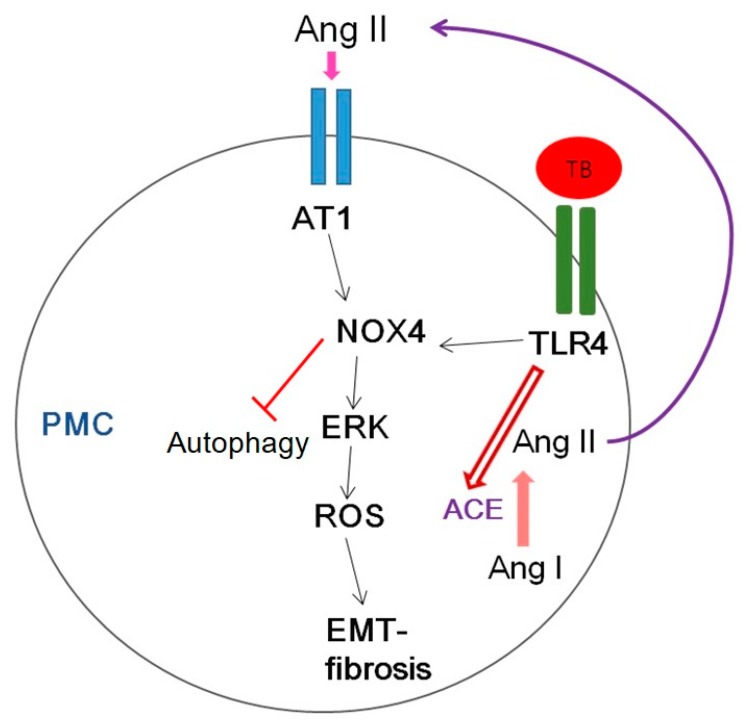
Schematic diagram of the signaling pathway relevant to HKMT-induced pleural fibrosis in pleural mesothelial cells (PMCs). AT1, Angiotensin II receptor type1; ROS, reactive oxygen species; EMT, epithelial mesenchymal transition; TB, tuberculosis; TLR4, Toll like receptor 4; ACE, angiotensin converting enzyme.

Figure 10 shows several potential signaling pathways through which NOX4 may contribute to tuberculous pleurisy.

Mycobacterium stimulated NOX4 expression via TLR4 and AT1R significantly induced ERK–ROS-collagen production and attenuated autophagic flux (Figure 4, Figure S1).

In human data, adults with tuberculous pleurisy had higher NOX4 levels in pleural effusion compared to adults with transudate. This result is in line with in vitro-human Met5A cell experiments, which showed that HKMT treatment leads to higher levels of NOX4 and subsequent EMT process. However, although this is fine for proof-of concept studies, these findings are limited to the normal human condition. Therefore, these finding should be expanded to include analysis in primary human mesothelial cells from patients with tuberculous pleurisy, to improve clinical relevance.

## 5. Conclusions

In conclusion, this study demonstrated that NOX4 signaling contributes to tuberculous pleural fibrosis and that NOX4 signaling may also regulate ERK–ROS signaling and EMT pathways. In the mouse experiment, the inhibition of NOX4 expression by SiNOX4 administration attenuated lung fibrosis and pleural inflammation. The clinical potential of NOX4 inhibitor ought to be further evaluated in future studies.

## Supplementary Materials

The following are available online at http://www.mdpi.com/2077-0383/8/1/116/s1, Figure S1: TLR4 inhibition and AT1 inhibition improve impaired autophagy caused by HKMT treatment. Figure S2: NADPH oxidase 4 (NOX4) levels in pleural effusion of adults with tuberculous pleurisy and transudates.

## Abbreviations

TB	tuberculosis
PMC	pleural mesothelial cell
HKMT	heat-killed mycobacterium tuberculosis
NOX4	NADPH oxidase
EMT	epithelial mesenchymal transition
ECM	extracellular matrix
ROS	reactive oxygen species
