# Supplementary material for: Inhibition of NADPH Oxidase 4 (NOX4) Signaling Attenuates Tuberculous Pleural Fibrosis
Source: J Clin Med. 2019 Jan 18;8(1):116. doi: 10.3390/jcm8010116 (PMC6351931; doi:10.3390/jcm8010116)
Supplement: Supplementary file 1 [file jcm-08-00116-s001.pdf]

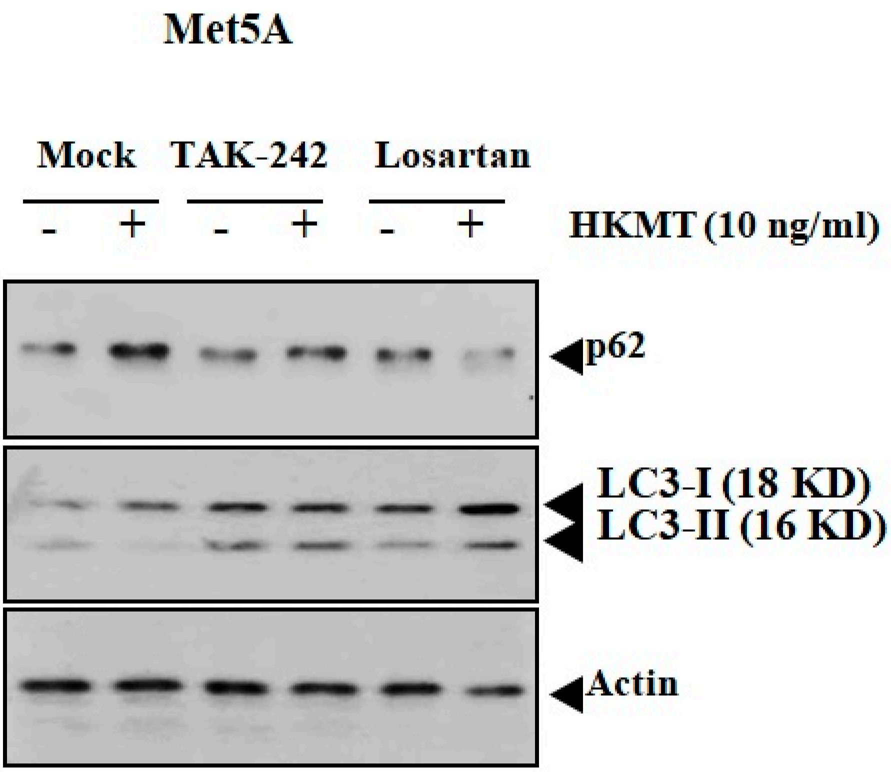

Figure S1. TLR4 inhibition and AT1 inhibition improve impaired autophagy caused by HKMT treatment. Western blotting analyses of LC3II and P62. TLR4, toll like receptor; AT1, angiotensin II receptor type I.

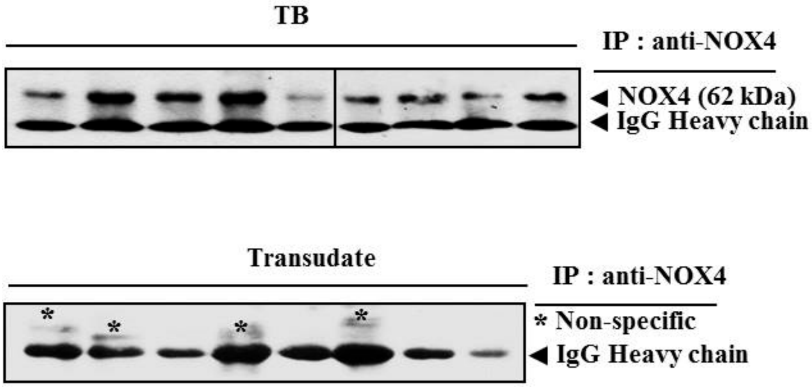

Figure S2. NOX4 levels in pleural effusion of adults with tuberculous pleurisy and transudates. Western blotting analyses of NOX4 were performed. IP, immunoprecipitation.
